# Migration and child health in Moldova and Georgia

**DOI:** 10.1186/s40878-017-0068-9

**Published:** 2018-02-08

**Authors:** Victor Cebotari, Melissa Siegel, Valentina Mazzucato

**Affiliations:** 1 UNICEF Office of Research-Innocenti, Piazza SS. Annunziata, 12, 50122 Florence, Italy; 2 Maastricht Graduate School of Governance, Maastricht University, Boschstraat 24, 6211 AX Maastricht, The Netherlands; 3 Department of Technology and Society Studies, Maastricht University, Grote Gracht 90-92, 6211 SZ Maastricht, The Netherlands

**Keywords:** Child health, Migration, Transnational families, Children left-behind, Moldova, Georgia

## Abstract

There is scarce empirical evidence on the relation between migration and child health in Moldova and Georgia—two post-Soviet countries with large out-migration flows in the region. This study uses nationally representative data collected in 2011–2012 in Moldova (*N* = 1601) and Georgia (*N* = 1193) to investigate how children’s health associates with five transnational characteristics: migrant and return-migrant household types, parental migration and parental divorce, maternal and/or paternal migration and caregiver’s identity, the duration of migration, and remittances. Findings show that, regardless of the transnational family setting, children of migrants have overall positive or no differing health compared to children in non-migrant households. However, significant gender differences are found in both countries. More often than not, Moldovan and Georgian girls are more at risk of having poorer health when living transnationally. These results add nuance to a field of research that has mainly emphasized negative outcomes for children in transnational care.

## Introduction

Following the collapse of the Soviet Union, approximately 21% of the Moldovan and 25% of the Georgian adult population had emigrated by 2010 (World Bank, 2011). Many of these migrants have children who often stay behind because of financial constraints and because of the transient nature of the work that migrants may encounter at their destinations. This process creates transnational families, those in which children and other family members experience a change in their living arrangements due to migration. Although the precise number of children who stay behind is not available, some sources estimate that approximatively 36 and 39% of all Moldovan and Georgian children, respectively, live in households where at least one member has migrated (Svintradze & Ubiria, 2007; Vanore, 2015). Over time, this situation has contributed to a normative discourse in Moldova and Georgia in which migration is associated with child abandonment and poor outcomes. In the face of this discourse, there is a need to better understand the relationship between transnational family formations and the well-being of children who stay behind.

Traditionally, research on parent–child separation has focused on the negative consequences that children may experience when separated from their parents because of extraordinary situations such as union dissolution or parental death (Amato & Cheadle, 2005; Corak, 2001). Not until recently scholars started to investigate how migration relates to child well-being (Cebotari, Mazzucato, & Appiah, 2017; Jordan & Graham, 2012; Wen & Lin, 2012) and child health (Cebotari, Mazzucato, & Siegel, 2017; Donato & Duncan, 2011; Gao et al., 2010; Stillman & McKenzie, 2012; Wen & Lin, 2012). In general, labor migration is found to result in large income gains that may help children to access better healthcare services at the place of origin (Amuedo-Dorantes & Pozo, 2011; Gerber & Torosyan, 2013; Kanaiaupuni & Donato, 1999). At the same time, the separation of children from migrant family members may affect children’s psychological (Mazzucato & Cebotari, 2017; Robila, 2012; Vanore, 2015), as well as physical health (Dreby, 2010; Stillman & McKenzie, 2012). To date, most studies of the effect of migration on children’s health have focused on Latin America, especially Mexico (Frank, 2005; Donato & Duncan, 2011; Kanaiaupuni & Donato, 1999), China (Gao et al., 2010; Wen & Lin, 2012) and Southeast Asia (Asis, 2006; Jordan & Graham, 2012), with some recent attention to African countries (Carling & Tønnessen, 2013; Cebotari, Mazzucato, & Siegel, 2017; Yabiku, Agadjanian, & Cau, 2012). Despite the surge of migration in former Soviet countries, there is almost no evidence concerning the relationship between migration and children’s health in the region.

Although research in other geographical areas provides substantial insight into the relationship between migration and child health, gaps remain. More often than not, studies rely on single-country analyses and employ simplistic notions of transnational family life associated with which parent migrates (mother, father or both) and whether or not they remit, thus hindering the effort to understand how multiple characteristics of transnational families affect children’s health. In particular, we know little about children’s health when different forms of parental absence coalesce, such as when parental migration is accompanied by divorce. What is more, studies often tend to focus exclusively on children in transnational care, without considering an appropriate control group of children in non-migrant households. This limits the opportunity to observe if variations in health are indeed due to migration.

This study builds on these gaps and attempts to understand the broader implications of labor migration on children; that is, to what extent does migration of household members relate to the health of children who remain in Moldova and Georgia? As such, we compare children in migrant and non-migrant households and aim to contribute to the literature in four ways. First, we investigate children who simultaneously experience migration and parental divorce, thereby adding marital discord as a transnational family arrangement when measuring children’s health. Second, we explore, on a large scale, the extent to which different forms of transnational family life relate to children’s health. We do so by employing different transnational characteristics such as the migrant or returned household types, the duration of migration, remittances, and parental migration and the caregiver type. Third, we include a gender perspective by investigating children’s health given these different forms of transnational family life. Recent studies have emphasized the need to distinguish between child’s gender when measuring children’s outcomes (Antman, 2011; Cortés, 2007; Vanore, 2015). Finally, we look at these characteristics in two places: Moldova and Georgia, thereby adding Eastern European cases to the literature. The comparison of two countries provides evidence of how migration associates with children’s health in a wider regional context. Overall, by engaging in this area of research, the results also address the dominant narrative of child vulnerability and poor well-being outcomes that pervades the discussion of children in migrant households in Moldova and Georgia.

## Background

### Study settings: Moldova and Georgia

Following the Soviet Union’s collapse in 1991, Moldova and Georgia became independent countries and began a difficult transition to democracy and market economy that continues relatively unabated today. In both countries, the economy and living standards declined steadily, especially during the first decade following independence. At the turn of the century, approximately 71% of Moldovans and 60% of Georgians were living below the national poverty line (Vanore, 2015). A number of factors contributed to this decline, including political instability, the loss of subsidies and access to the markets of the Soviet era, hyperinflation and a dramatic drop in economic outputs of both countries. These problems were amplified by separatist movements and the outbreak of civil wars in the Transnistrian region of Moldova and in Abhazia and South Osetia regions of Georgia. Over the last two-and-a-half decades, active and passive forms of warfare have persisted and there is yet to be reconciliation between the separatist regions and central state authorities.

The combination of conflict and economic difficulties encouraged sustained out-migration flows from Moldova and Georgia. The main destinations of Moldovans are Russia, Ukraine, Italy, and Romania while Georgian migrate mostly to Russia, Greece, Turkey, the United States, and Germany (World Bank, 2011). The majority of Moldovans and Georgians who migrate are working age, skilled individuals: this is true for both men and women although more men migrate eastwards to Russia whereas more women migrate westwards to meet the growing demand for home and elderly care services in the European Union countries (Labadze & Tukhashvili, 2013; UNICEF, 2008).

In Moldova and Georgia, family systems are characterized by norms in which the nuclear or the extended family are responsible with the daily care of children whose parents migrated. Although the function of care stays within the family, there are some nuances worth noting. Specifically, Moldovan households are more nuclear whereas Georgian households are often multigenerational (Vanore, 2015). It is also more common for children in Moldova to experience the migration of both parents, which results in more cases of Moldovan children who live without an adult caregiver or who often move to another residency to stay with a family relative (Cebotari, Siegel, & Mazzucato, 2016; UNICEF, 2016).

### The absence of family members and Children’s health

Money and time are crucial resources that family members can provide for children (Suárez-Orozco, Todorova, & Louie, 2002). When family members migrate, the stress and distance associated with their departure may have a deleterious effect on the psychological health (Mazzucato & Cebotari, 2017; Vanore, 2015) and the physical health (Dreby, 2010; Stillman & McKenzie, 2012; Wen & Lin, 2012) of children in transnational care. However, children may also be better off when household members migrate because migration generally yields economic benefits such as remittances that can be used to invest in children’s health (Amuedo-Dorantes & Pozo, 2011; Donato & Duncan, 2011; Gerber & Torosyan, 2013). Thus, there may be advantages and disadvantages to children’s health when household members migrate.

The main consequence of migration for children is that they often live separated from one or both parents for an extended period of time. Importantly however, migration is not the *only* reason parents and children may be separated, and other events like divorce factor in as well. Traditionally, the consequences of union dissolution have been analyzed by family studies in Western contexts, revealing the emotional and behavioral disadvantages for children living in single-parent families compared with children in two-parent families (Amato & Cheadle, 2005; Corak, 2001). Until recently, the effects of parental divorce in the context of migration have received little scholarly attention. In one of the few studies that examines marital relationships in the context of migration, Nobles (2011) observed that separation because of divorce affects Mexican children more negatively than separation because of migration. In Ghana, Mazzucato and Cebotari (2017) found deleterious psychological health among children whose parents migrated and were divorced but not when parents migrated and were together. Similarly, in Ghana and Nigeria, children of divorced migrant parents were less likely than children in non-migrant families to rate their health as good (Cebotari, Mazzucato, & Siegel, 2017). Furthermore, in Malawi, Carling and Tønnessen (2013) observed that children living with both parents and children with migrant fathers in stable relationships had better physical health and nutritional outcomes than children with divorced parents.

In general, then, migration and divorce seem to pose different experiences for children, and the two combined often reflect on children’s health more negatively. The different outcomes associated with migration, divorce, or both are likely explained by specifics in these forms of absence. Intrinsically, migration is often motivated by a desire to improve the lives of children and of other family members who stay at origin (Nobles, 2011). In cases of divorce however, the well-being of children does not always motivate the intention to migrate. For instance, migration was found to be a way to escape a problematic marriage for some Filipina wives (Constable, 2003). Migration also has the potential to strain marital relationships (Pribilsky, 2004), especially when women migrate alone, or when migrant spouses have different ethnic backgrounds (Kulu & González-Ferrer, 2014). When migration and divorce coalesce, parents may lack the necessary resources to invest in children, particularly when the divorced parents re-marry and have children in those new unions (Dreby, 2010). These processes were found to affect children’s living arrangements, including the ability to adapt to new authority figures (Suárez-Orozco et al., 2002) and to the loss in the remitting flow from divorced migrant parents (Abrego, 2009). The dynamics of living with migration and divorce point to the need to study the effects of marital dissolution on children in transnational care.

Most large-scale studies on transnational families restrict their focus to children whose migrant parents are in a stable union in order to isolate the sole effects of migration on children’s health (Donato & Duncan, 2011; Wen & Lin, 2012). These studies show that the migration of household members is often a household strategy to increase the welfare and well-being of members who stay behind. A stream of research also looks at the economic benefits of migration and shows that remittances sent home are often used to improve children’s health (Asis, 2006; Donato & Duncan, 2011; Frank, 2005) and provide better access to healthcare services (Lindstrom & Muñoz-Franco, 2006). Furthermore, evidence shows that in Mexico, the health of infants in migrant remitting households is improved compared to that of infants in non-migrant households (Frank, 2005). Similarly, in the Philippines and Georgia, remittances from migrant household members were found to positively associate with children’s health (Asis, 2006; Gerber & Torosyan, 2013). The effect of remittances on health also has a positive spillover effect on children in non-migrant households because better access to healthcare for children in migrant households reduces the emergence and transmission of preventable diseases within the larger community (Kanaiaupuni & Donato, 1999).

However, these and other studies offer little insight into differences in health for children in households whose members migrated but returned. Understanding the health implications of return migration is important because prior research notes difficulties for former migrants upon return. For example, studies in Mexico found that migrants who return are less healthy, which may affect children’s health prospects as well (Donato & Duncan, 2011). Former migrants and their children may also be disadvantaged because they no longer have regular access to financial capital from abroad or to the health services that capital purchases (Dreby, 2010). Furthermore, returned migrants have often poor knowledge of the public health programs and a less stable employment history in the country of origin, which, in contrast to non-migrant families, may limit the access of family members to public-based health insurance schemes (Donato & Duncan, 2011; UNICEF, 2016).

The literature on parental absence because of migration generally indicates harmful effects of parental separation although the magnitude of these effects appears to vary according to which parent has migrated (Cortés, 2007; Jordan & Graham, 2012; Parreñas, 2005; Schmalzbauer, 2004). According to these studies, the mother’s absence often has greater behavioral consequences and leads to problems for children. When fathers migrate, mothers generally take over the caregiving responsibilities within the household, whereas when mothers migrate, fathers often rely on other household members to care for the children (Cortés, 2007). More recently, research has emphasized the need to consider parental migration in relation to the caregiver of the child who stay behind. Studies conducted in Moldova (Vanore, 2015) and in Ghana and Nigeria (Cebotari, Mazzucato, & Siegel, 2017) observed no differences in children’s health when mothers migrate while children stay in the care of the father. Another study found improved child health when fathers migrate and children remain in the care of mothers in Mozambique (Yabiku et al., 2012). More evidence is needed from large-scale studies to assess the role of parents and other household members as caregivers for children in transnational families, as this has only started to be investigated in relation to children’s health.

Previous research has also detected significant gender differences when measuring the health of children living in migrant households. In China, for example, girls were observed to be more at risk for unhealthy behaviors, such as smoking and drinking, and boys were on average more overweight and less physically fit than children living in non-migrant families (Gao et al., 2010). In Moldova, boys were observed to have more abnormal psychological health when fathers were abroad and when they were cared for by non-parental caregivers (Vanore, 2015). Some of these gender differences may be caused by different gender roles in countries of origin. Different values and norms related to family and care may shape the behavior of child rearing in migrant sending communities (Kulu & González-Ferrer, 2014). For instance, Cortés (2007) observed that girls have more physical chores at home compared to boys, even when the household’s economic situation is improved by remittances. In Mexico, however, studies concluded that traditional gender inequality is reduced in migrant households, especially when household members return from abroad (Antman, 2011). More studies are warranted to explore the gender differences in children’s health when household members migrate.

### Hypotheses

The extant research reviewed above encompasses many conditions that influence children’s health. Collectively, it suggests that migration does not always result in worse health for children who stay in the home country. Based on this research, we advance several hypotheses.

Hypothesis 1. We expect that the presence of parental divorce, whether accompanied by migration or not, will affect children’s health more negatively compared with children living in non-migrant households whose parents are in a stable marital union.

Hypothesis 2. We expect that children living in households with current or returned migrants will have more means to invest in children’s health, resulting in better health compared with children in non-migrant households.

Hypothesis 3. We expect that maternal migration and the migration of both parents, regardless of who the caregiver is, will have a more negative effect on children’s health when compared with children in non-migrant households.

Hypothesis 4. Because longer periods of absence may result in less contact among households members, we expect the duration of migration to be negatively associated to children’s health.

Hypothesis 5. We expect the absence of remittances to relate more negatively, whereas the presence of remittances to associate more positively with the health of children in transnational care, compared with children living in non-migrant households.

Hypothesis 6. We expect that girls are more at risk to have poorer health compared to boys when living in different transnational forms of care.

## Methods

### Data and sample

Data used in this study are from two nationally representative, cross-sectional, and comparable large-scale household surveys conducted between 2011 and 2012 in Moldova and Georgia. The surveys in both countries were drawn from a random stratified sample of households in which at least one child below the age of 18 resided. Survey questionnaires were administered to eligible households following the random walk method. In Moldova, the sampling frame was drawn from the Labor Force Survey (LFS), which is based on the population census and the updated lists of electricity consumers. In Georgia, the sampling frame was created based on the national electoral districts. The country’s regions were divided into six strata each based on settlement size and type. The strata were then assigned primary sampling units (PSUs) based on electoral districts. Within each PSU, households were selected following a stratified random sampling. Following the data collection, sampling weights were designed to allow for adjustments in the distribution of sample characteristics across the entire population and make the results representative at the national level. Specifically, in Moldova sampling weights were designed based on most recent population characteristics available in the LFS sampling records, whereas in Georgia, sampling weights were derived from the distribution of population characteristics collected from a listing exercise among all contacted households in selected electoral districts. Information on age-groups, gender, education, and region was used to define the weights and to test the representativeness of data at the national level.

Both surveys covered all administrative regions except for the breakaway regions of Transnistria in Moldova and Abkhazia and South Ossetia in Georgia. The collection of data followed the ethical standards of the International Code on Market and Social Research for Opinion and Market Research (ESOMAR). Data were collected in migrant and non-migrant households in which at least one child was living when the survey took place. Migrant households were considered those in which at least one household member was abroad for 3 months or more at the time of the survey. All respondents gave informed consent to participate and the questionnaire was administered in local languages. The questionnaire was primarily administered to the head of the household although child-specific information was collected from the primary caregiver of each child. From these caregivers, we obtained information on 1601 Moldovan and 1193 Georgian children aged between 10 and 18. These samples comprise children in both migrant and non-migrant households but do not include children whose parents were deceased.

### Measures

For this analysis, the dependent variable is the child’s health status as assessed by the main caregiver at the time of the survey. This assessment is measured by the question, “Compared to other children of this age, would you say [child]‘s health is *much better, better, neither better nor worse*, *worse*, or *much worse*?” The responses were further dichotomized and received a score of 1 if they were either *much better* or *better* while the remaining response categories received a score of 0. We dichotomize this measure to better capture the positive and negative variations in the outcome. This rationale for dichotomization has been used and validated in previous research assessing child well-being, including health (Carling & Tønnessen, 2013; Jordan & Graham, 2012).

A subjective measure of child health was chosen because it assesses the health in relation to children’s peers in a given community, which is optimal when measuring the effects of migration and health with cross-sectional data. Although the assessment of children’s subjective health by caregivers may contain reporting errors, research indicates that such responses accurately predict children’s mortality and the use of medical healthcare (Idler & Benyamini, 1997). A more recent study by Donato and Duncan (2011) used a similar subjective measure to assess children’s health in Mexico, producing robust evidence. Nevertheless, because caregivers’ reports may reflect subjective worries that may lead to inflated perceptions of a child’s health, our analysis also controls for the caregiver’s self-assessed level of happiness on a ten-point Likert scale (Vanore, 2015) and for the quality of the child-caregiver relationship, as reported by the caregiver (Jordan & Graham, 2012).

Five migration indicators were constructed for this analysis. Using a set of questions detailing individuals’ current and past migration histories, the first variable indicates three *types of households* related to migration: non-migrant, households in which at least one member is abroad, and households with at least one member who migrated and returned (on the condition that there is no other member who is currently migrant). The second indicator looks at the *type of separation* in relation to parental absence–whether children have both parents in the country and in a marital union, have both parents in the country and divorced/separated, have one or both parents abroad and in a marital union, and have one or both parents abroad and divorced/separated. The third indicator accounts for *which parent has migrated and the identity of the caregiver* when parents are away–both parents are non-migrants, the father is migrant and the mother is the caregiver, the mother is migrant and the father is the caregiver, both parents are migrants and a grandparent is the caregiver and both parents are migrants and the child is cared for by someone else. A fourth indicator measures the *duration of migration* of household members. This indicator distinguishes between children of non-migrants, children whose household members stay short periods abroad (irregular and seasonal migration), and children whose household members stay longer periods abroad (1 year or more). Finally, we look at the availability of *remittances* by measuring whether children live in households that receive monetary or in-kind remittances from the migrants – *yes, no*.

Additional indicators were included to control for child-, household- and caregiver-level characteristics. The child-level controls included age and gender. Household-level controls included a binary variable of the present living conditions of the household, where 1 indicates good living conditions. Additional household-level controls included the total number of children and a measure of housing quality, which was determined by dividing the number of individuals by the number of rooms in the house. The caregiver-level characteristics comprised the caregiver’s years of completed education, the reported quality of the child-caregiver relationship, and the level of reported happiness on a ten-point Likert scale in which higher scores indicate greater happiness. Finally, we included a region indicator because there may be geographical differences in assessing children’s subjective health in the two countries.

### Analysis

This study fitted binary logistic regressions to test the established hypotheses. Modelling transnational migration in relation to children’s health is complicated by endogeneity problems. The decision to migrate has multiple rationales and it is possible that children’s health may have influenced migration if migrants’ departure was motivated by a desire to improve the well-being of children. It is difficult to account for endogeneity problems when using modelling techniques with cross-sectional data. The issue of migrant selectivity also requires attention. Migration involves material resources and those who are able to provide better resources for children at home may also be more likely to migrate. Furthermore, migration generally requires good health and children in transnational care may share with their migrant household members a latent predisposition for better health. Previous research has used instrumental variables or employed a randomized “natural” experiment to control for the selectivity of migration in relation to children’s health (for instance, Stillman & McKenzie, 2012). However, employing reliable instruments without affecting the dependent variable is problematic, especially when working with cross-sectional data (Adams, Haas, Jones, & Osili, 2012). In the absence of instrumental designs and considering the data at hand, the second best strategy is employed. Specifically, a variety of transnational family characteristics and other observables described above are included to partially address the selectivity issues. However, it is difficult to account for all possible conditions that affect migrant selectivity and child health and we invite readers to keep this in mind as they explore the results.

The analysis was conducted in two stages. In the first stage, we examined whether there is a relationship between children’s reported health and parental absence that involves three dimensions – migration, marital dissolution, and both migration and marital dissolution. A stepwise approach is employed in which we controlled for children’s characteristics, for household- and caregiver-level indicators, and for the region variable.

In a second stage, we tested whether the effects of parental migration observed in the first stage of analysis remain valid if we consider different transnational characteristics such as the migrant or return-migrant household, duration of migration, remittances, and the identity of the caregiver in relation to parental migration. To isolate migration’s role as a unique form of absence, this stage of analysis excludes those children whose parents are away and divorced (*N* = 207 in Moldova; *N* = 175 in Georgia). Bivariate means comparison tests identified no differences in the means of the key indicators between the excluded and retained observations, suggesting that cases were missing at random.

The analyses do not include different migration characteristics in one model for two reasons. First, each transnational characteristic captures specific effects of migration and we model them separately without the interfering effects of other migration characteristics. Second, each migration indicator compares children in migrant and non-migrant households. This makes transnational characteristics collinear because they each contain a reference category of children living without a migration experience. The reference category of children in non-migrant households is consistently included in all migration indicators to observe whether the effects apply to a larger population of children than those living transnationally.

Interactions of gender and key measurements were also tested and included in all models, helping to establish whether there are gender-related effects when examining the specifics of migration. Only those interactions with significant effects were included in the tables presenting results. All regression results are presented as odds ratios with confidence intervals. Sampling weights were applied in all models to adjust for the eventual errors in the sampling design and to render results representative at the population level.

## Results

Table 1 shows the descriptive characteristics of the sample. For brevity, we present only the evidence pertaining to the dependent variable and main predictors. In the sample populations, fewer children are reported to have good/very good health in Moldova (40.69%) than in Georgia (59.19%). Numbers under the column headings indicate that approximately 54.97% and 51.42%, respectively, of Moldovan and Georgian children live in non-migrant households. Another 28.11% of Moldovan children and 39.11% of Georgian children live in households with members abroad. The proportion of children living in households with returned migrants is 16.92% in Moldova and 9.47% in Georgia. Furthermore, 12.93% (8.62% + 4.31%) and 14.76% (8.55% + 6.12%) of Moldovan and Georgian children, respectively, have divorced parents, either in the country or abroad. Unfortunately, we are unable to establish whether migration precipitates or is precipitated by divorce because of the lack of the information of the timing of divorce in the data. However, a previous study in Moldova and Georgia found a positive relationship between migration and divorce, especially among female migrants (Vanore, 2015). The percentage of children with parents abroad in a stable marital relationship was 19.93% in Moldova and 21.88% in Georgia.

**Table 1:** Means/percentages (Standard Deviations) of dependent and independent variables

	Moldova^a^	Georgia^b^
Variables	Percentage/mean (SD)	Percentage/mean (SD)
Child’s health is good/very good	40.69	59.19
Type of separation	100	100
Parents in the country and in a marital union	67.15	63.45
Parents in the country and divorced	8.62	8.55
Parent(s) abroad and in a marital union	19.93	21.88
Parent(s) abroad and divorced	4.31	6.12
Household type	100	100
Non-migrant household	54.97	51.42
Migrant household	28.11	39.11
Returned migrant household	16.92	9.47
Duration of migration	100	100
Non-migrant household	71.89	60.89
Short period abroad	23.01	25.34
Long period abroad	5.10	13.77
Parental migration and caregiver’s identity	100	100
Non-migrant: live with both parents	77.51	74.65
Father migrant: mother caregiver	11.39	15.19
Mother migrant: father caregiver	7.07	7.30
Both parents abroad: grandmother caregiver	3.17	2.47
Both parents abroad: other caregiver	0.87	0.39
Remittances	100	100
Non-migrant household	71.89	60.89
Migrant household: no	10.27	11.22
Migrant household: yes	17.84	27.89
Child is female	48.07	46.04
Age (in years)	14.3 (2.59)	13.44 (2.40)
Caregiver years of education	10.85 (2.58)	12.88 (2.68)
Good living conditions	74.35	70.92
Nr. of People *per* Nr. of Rooms	1.31 (0.72)	1.34 (0.87)
Number of children in household	1.83 (1.04)	1.84 (0.82)
Distant relationship with the caregiver	11.50	7.36
Happiness status caregiver	6.52 (2.11)	6.52 (2.2)
Region Moldova	100	
Centre	38.04	
Chisinau	5.84	
North	28.17	
South	27.95	
Region Georgia		100
Tbilisi		26.94
Guria		2.87
Adjara		11.49
Shida-Kartli		5.82
Kaxeti		6.06
Kvemo-Kartli		12.66
Samegrelo		11.57
Imereti		16.30
Samcxe-Javaxeti		3.26
Mcxeta-Tianeti		3.03

*Notes:* Standard deviations in parentheses

^a^All indicators in the Moldova sample have 1394 observations, except for the *type of separation* that has 1601 observations

^b^All indicators in the Georgia sample have 1018 observations, except for the *type of separation* that has 1193 observations

Of those children with migrant parents, the greatest proportion had fathers abroad, with mothers as caregivers. Children with mothers abroad and in the care of fathers accounted for 7.07% in Moldova and 7.30% in Georgia. Furthermore, children with both parents abroad and cared for by grandparents represented 3.17% and 2.47% of the sampled populations in Moldova and Georgia, respectively. The proportion of children cared for by others (non-parental/non-grandparental) is insignificant in both countries. It is therefore mentioned here but not included in the final analysis.

Further, a short period of migration is more common than longer periods of migration in both countries. We also noted that more children live in migrant households who receive remittances in Georgia than in Moldova (27.89% compared with 17.84%). The percentages of children living in migrant households without remittances are 10.27% and 11.22%, respectively, in Moldova and Georgia. The relatively high proportion of children living in migrant households without remittances may reflect irregular remitting channels and the commonality of seasonal migration, which is less lucrative in terms of remittances.

A series of binary logistic models were fitted to estimate associations between different transnational family indicators, the specified controls and children’s health. The first binary logistic analysis assesses the relation between parental absence – through migration, marital dissolution, or both migration and marital dissolution – and the reported health status of children in migrant and non-migrant households. Table 2 presents the stepwise models of this analysis in which the baseline models summarize the effects of different dimensions of parental absence and children’s characteristics in relation to child health in Moldova and Georgia (Models 1 and 4). The subsequent models add household- and caregiver-level indicators (Models 2 and 5) and the region variable (Models 3 and 6).

**Table 2:** Parental migration, marital status and children’s health

	Moldova						Georgia					
	Model 1		Model 2		Model 3		Model 4		Model 5		Model 6	
Type of separation (ref. parents in the country and in a marital union)												
Parents in the country and divorced	0.81	[0.21,3.09]	0.95	[0.23,3.85]	0.91	[0.21,3.85]	0.79	[0.15,3.98]	0.96	[0.18,5.05]	0.98	[0.15,6.074]
Parent(s) abroad and in a marital union	3.25^**^	[1.34,7.91]	3.47^**^	[1.41,8.57]	3.26^*^	[1.28,8.32]	4.10^*^	[1.04,9.16]	3.28^*^	[0.80,8.89]	4.85^*^	[1.16,9.17]
Parent(s) abroad and divorced	1.60	[0.27,9.54]	1.99	[0.32,12.3]	1.66	[0.27,9.99]	0.39	[0.04,3.62]	0.43	[0.07,4.15]	0.42	[0.04,3.72]
Female ^*^ parent(s) abroad and in a marital union							0.38^*^	[0.15,0.93]	0.43^*^	[0.17,1.07]	0.35^*^	[0.14,0.88]
N	1601		1601		1601		1193		1193		1193	
Wald χ^2^	17.61		45.46		88.99		32.51		38.34		98.65	
McFadden’s pseudo-R^2^	0.11		0.10		0.11		0.15		0.17		0.16	
Log-pseudolikelihood	−2345.12		−2838.91		−2479.67		−681.83		−675.93		−593.72	

*Notes.* Reported results are odds ratios; 95% confidence intervals in brackets. Models 1 and 4 include the variable of interest (the type of separation) and child-level characteristics (gender and age). Models 2 and 5 add household- and caregiver-level characteristics (caregiver’s education, living and housing conditions, number of children, child-caregiver relationship, and caregiver’s happiness status. Models 3 and 6 add the region indicator

* *p* < 0.05, ** *p* < 0.01, *** *p* < 0.001

In both countries, models show that children with migrant parents in a stable marital relationship are better off in terms of health than children in non-migrant households whose parents are also in a stable union. Notably however, children with divorced parents, whether in the country or abroad, do not appear to experience significantly poorer health than children of non-migrants. Based on this evidence, we reject Hypothesis 1. Further, interaction terms are included to examine whether the effects of different dimensions of parental absence vary by child’s gender. In Georgia, we find that being a girl reduces the overall positive effects of parental migration and a stable marital relationship on child health (Models 4–6, Table 2). This finding gives initial support to Hypothesis 6 and may point to persistent gender inequalities, described in Georgia (Vanore, 2015) and in other contexts (Cebotari, Mazzucato, & Appiah, 2017; Gao et al., 2010; Cortés, 2007; Parreñas, 2005).

The second analysis disaggregates the migration component into four characteristics: transnational household type, duration of migration, parental migration and caregiver’s identity, and remittances (Tables 3 and 4). Each of these transnational settings is separately regressed on the full set of controls. The data show that both Moldovan (Model 7, Table 3) and Georgian children (Model 11, Table 4) in migrant households are more likely to have better reported health than children in non-migrant households. Additionally, Moldovan children living in returned migrant households display better health, whereas the reported health of children in Georgia in returned migrant households does not differ significantly from the reported health of children of non-migrants. Hypothesis 2 is, therefore, confirmed. To examine whether the effects of migration or return migration vary by children’s gender, we estimate interactions in the models. In Moldova and Georgia, the interaction between a child’s gender and living in a migrant household is negatively associated with girls’ reported health. This evidence gives additional support to Hypothesis 6 and points to patterns of investments by migrant and return-migrant households that differ significantly along the dimension of gender.

**Table 3:** Different transnational family characteristics and children’s health: Moldova

	Model 7 Household type		Model 8 Duration		Model 9^a^ Parental migration and caregiver		Model 10 Remittances	
Household type (ref. non-migrant household)								
Migrant household	3.09^*^	[1.28,7.47]						
Returned migrant household	2.93^*^	[1.01,8.48]						
Female ^*^ migrant household	0.52^*^	[0.29,0.91]						
Duration of migration (ref. non-migrant household)								
Short period abroad			1.86	[0.74,4.62]				
Long period abroad			3.18^*^	[1.05,7.42]				
Parental migration and caregiver’s identity (ref. non-migrant: live with both parents)								
Father migrant: mother caregiver					3.55^**^	[1.64,8.71]		
Mother migrant: father caregiver					2.11	[0.46,9.60]		
Both parents abroad: grandmother caregiver					4.33	[0.69,9.82]		
Female ^*^ Father migrant: mother caregiver					0.41^*^	[0.20,1.63]		
Remittances (ref. non-migrant household)								
Migrant household: no							2.81	[1.07,5.58]
Migrant household: yes							2.15	[0.79,5.80]
Child is female	1.59^**^	[1.14,2.22]	1.40^*^	[1.04,1.87]	1.39^*^	[1.04,1.85]	1.43^*^	[1.06,1.91]
Age (in years)	1.04	[0.99,1.09]	1.04	[0.99,1.10]	1.05	[0.99,1.10]	1.04	[0.99,1.09]
Caregiver years of education	1.02	[0.97,1.08]	1.02	[0.97,1.08]	1.02	[0.97,1.08]	1.02	[0.97,1.07]
Good living conditions	1.55^**^	[1.12,2.14]	1.59^**^	[1.15,2.19]	1.47^*^	[1.05,2.04]	1.58^**^	[1.14,2.19]
Nr. of People *per* Nr. of Rooms	0.86	[0.70,1.07]	0.86	[0.69,1.06]	0.87	[0.69,1.09]	0.85	[0.68,1.05]
Number of children in household	1.08	[0.94,1.24]	1.07	[0.93,1.23]	1.07	[0.93,1.23]	1.07	[0.93,1.23]
Distant relationship with the caregiver	1.20	[0.54,2.67]	1.16	[0.51,2.65]	0.82	[0.31,2.16]	1.26	[0.56,2.81]
Happiness status caregiver	1.09^*^	[1.01,1.16]	1.09^*^	[1.02,1.16]	1.09^*^	[1.02,1.17]	1.09^*^	[1.01,1.16]
Chisinau (ref. Centre)	1.85^*^	[1.06,3.22]	1.81^*^	[1.03,3.15]	1.96^*^	[1.11,3.46]	1.82^*^	[1.05,3.16]
North	1.19	[0.87,1.62]	1.20	[0.88,1.64]	1.24	[0.90,1.69]	1.20	[0.88,1.64]
South	1.87^***^	[1.40,2.49]	1.86^***^	[1.40,2.48]	1.92^***^	[1.43,2.57]	1.88^***^	[1.41,2.51]
N	1394		1394		1394		1394	
Wald χ^2^	64.53		68.78		76.92		79.92	
McFadden’s pseudo-R^2^	0.11		0.09		0.08		0.09	
Log-pseudolikelihood	−2854.24		−2039.54		−1910.48		−2852.94	

*Notes:* Reported results are odds ratios; 95% confidence intervals in brackets

^*^p < 0.05, ^**^ *p* < 0.01, ^***^p < 0.001

^a^Model 9 omits children in the category ‘both parents abroad: other caregiver’, as this category contained too few observations for inclusion in analysis

**Table 4:** Different transnational family characteristics and children’s health: Georgia

	Model 11 Household type		Model 12 Duration		Model 13^a^ Parental migration and caregiver		Model 14 Remittances	
Household type (ref. non-migrant household)								
Migrant household	3.58^*^	[1.19,9.73]						
Returned migrant household	5.28	[0.48,6.21]						
Female ^*^ migrant household	0.38^**^	[0.18,0.79]						
Duration of migration (ref. non-migrant household)								
Short period abroad			1.61	[0.44,5.80]				
Long period abroad			8.74^**^	[1.93,19.56]				
Female ^*^ Long period abroad			0.19^**^	[0.06,0.57]				
Parental migration and caregiver’s identity (ref. non-migrant: live with both parents)								
Father migrant: mother caregiver					7.39^*^	[1.31,16.77]		
Mother migrant: father caregiver					2.05	[0.17,21.01]		
Both parents abroad: grandmother caregiver					0.71	[0.01,6.76]		
Female ^*^ Father migrant: mother caregiver					0.23^*^	[0.07,0.72]		
Remittances (ref. non-migrant household)								
Migrant household: no							1.67	[0.40,6.97]
Migrant household: yes							2.82^*^	[1.01,7.85]
Female ^*^ Migrant household: yes							0.39^**^	[0.20,0.76]
Child is female	1.79^**^	[1.18,2.71]	1.54^*^	[1.05,2.26]	1.53^*^	[1.03,2.28]	1.59^*^	[1.07,2.38]
Age (in years)	0.99	[0.92,1.08]	0.99	[0.91,1.07]	0.99	[0.91,1.08]	0.99	[0.91,1.07]
Caregiver years of education	1.03	[0.95,1.11]	1.03	[0.95,1.11]	1.03	[0.95,1.11]	1.02	[0.95,1.10]
Good living conditions	1.14	[0.73,1.79]	1.18	[0.75,1.85]	1.18	[0.74,1.88]	1.17	[0.74,1.83]
Nr. of People *per* Nr. of Rooms	0.99	[0.81,1.21]	0.98	[0.80,1.19]	1.01	[0.82,1.24]	0.97	[0.79,1.18]
Number of children in household	0.88	[0.68,1.15]	0.89	[0.68,1.15]	0.86	[0.66,1.13]	0.89	[0.69,1.15]
Distant relationship with the caregiver	1.50	[0.48,4.66]	1.49	[0.48,4.68]	1.31	[0.35,4.84]	1.50	[0.48,4.66]
Happiness status caregiver	1.00	[0.92,1.10]	1.01	[0.92,1.10]	1.02	[0.92,1.10]	1.01	[0.92,1.10]
Guria (ref. Tbilisi)	0.73	[0.21,2.48]	0.68	[0.20,2.25]	0.67	[0.21,2.12]	0.73	[0.21,2.47]
Adjara	2.11^*^	[1.16,3.84]	2.13^*^	[1.17,3.88]	2.31^**^	[1.23,4.32]	2.07^*^	[1.13,3.77]
Shida-Kartli	4.64^**^	[1.78,12.0]	4.72^**^	[1.83,12.16]	4.73^**^	[1.82,12.2]	4.63^**^	[1.80,11.90]
Kaxeti	4.76^**^	[1.86,12.2]	4.69^**^	[1.82,12.06]	6.20^***^	[2.39,16.11]	4.59^**^	[1.78,11.87]
Kvemo-Kartli	3.25^**^	[1.60,6.60]	3.26^***^	[1.65,6.43]	3.34^***^	[1.66,6.68]	3.16^***^	[1.60,6.22]
Samegrelo	15.5^***^	[6.30,38.5]	15.35^***^	[6.23,37.80]	15.40^***^	[6.25,37.92]	15.14^***^	[6.15,37.28]
Imereti	0.80	[0.46,1.41]	0.79	[0.46,1.38]	0.83	[0.47,1.46]	0.78	[0.45,1.36]
Samcxe-Javaxeti	1.65	[0.60,4.52]	1.61	[0.61,4.46]	1.56	[0.53,4.42]	1.62	[0.60,4.39]
Mcxeta-Tianeti	0.75	[0.26,2.07]	0.75	[0.27,2.10]	0.88	[0.30,2.57]	0.77	[0.27,2.15]
N	1018		1018		1018		1018	
Wald χ^2^	106.86		105.83		109.45		106.85	
McFadden’s pseudo-R^2^	0.18		0.15		0.19		0.18	
Log-pseudolikelihood	−576.32		−502.93		−434.76		−523.93	

*Notes:* Reported results are odds ratios; 95% confidence intervals in brackets

^*^ *p* < 0.05, ^**^ *p* < 0.01, ^***^ *p* < 0.001

^a^Model 13 omits children in the category ‘both parents abroad: other caregiver’, as this category contained too few observations for inclusion in analysis

In Moldova and Georgia, children are likely to be in better health when living in households in which relatives remain abroad for extended periods (Model 8, Table 3; Model 12, Table 4). There is no difference in reported health between children living in migrant and non-migrant households when migrantts remain abroad for shorter periods of time. Hypothesis 4 is thus rejected. However, Georgian girls were less likely to be assessed with better health when relatives stayed longer periods abroad. This finding provides an indication that while caregivers generally assess children’s health more positively, they nevertheless may view longer periods of absence as particularly harmful for girls.

To assess whether children’s reported health varies according to the migration status of parents, we regressed categories measuring parental migration and caregiver’s identity (non-migrant, migrant fathers and caregiver mothers, migrant mothers and caregiver fathers, and both parents migrants and caregiver grandparents) on the full set of controls (Model 9, Table 3; Model 13, Table 4). In both countries, children whose mothers are caregivers when fathers migrate are more likely to have better health than children living in non-migrant households. Again, gender differences are observed, with girls less likely to be assessed with better health when fathers migrate and mothers are caregivers in the two contexts in focus of this study. Notably, in both countries, no significant effects are observed when mothers migrate and fathers are caregivers or when both parents migrate and children are cared for by grandparents. Consequently, we reject Hypothesis 3.

The final part of the analysis considers whether the presence or absence of remittances is linked to children’s health. In Moldova, results show no differences in the health assessments of children in transnational and non-transnational care when households receive or do not receive remittances (Model 10, Table 3). In Georgia, however, children in households who receive remittances are more likely to be assessed with better health (Model 14, Table 4). This evidence gives partial support to Hypothesis 5. In Georgia, there is also evidence that girls in migrant households who receive remittances are generally less likely to have better health. In this country context, girls may benefit less from improved material well-being of households from remittances.

## Discussion

The separation of children from family members because of migration is increasingly common in developing countries such as Moldova and Georgia. As migration unfolds, there are concerns that living in transnational care may result in negative consequences for children. Although the migration of household members may come at the cost of separation and loss, it can also bring advantages, such as the potential for development through remittances. The empirical evidence to date, however, is mixed, with no studies looking at the health of children in transnational care in Moldova and Georgia. In line with the emergent field of transnational family studies, this analysis adds four reflections to the current scholarship. First, this study is among the first to distinguish between migration and marital discord as forms of separation when assessing children’s health. Second, the life of children in transnational care is complex and we acknowledge this complexity by investigating different transnational family forms in relation to children’s health. Third, our findings integrate a gender perspective by comparing boys and girls when measuring the outcome. Finally, this study adds evidence from two Eastern European contexts to a body of research that is scarcely represented in the region. We discuss these contributions below.

The findings of this study contribute to an emergent stream of research that looks at migration and marital dissolution and how this relation may affect children. Previous research found negative health outcomes for children when parents are abroad and divorced (Carling & Tønnessen, 2013; Cebotari, Mazzucato, & Siegel, 2017). Our study runs counter to such an assumption and shows that children whose parents are divorced and away do not have differing health from that of children in non-migrant households. However, this finding does not necessarily indicate that the divorce narrative in the context of migration is totally misplaced. Over the past decade, the boom in communication technologies has made transnational communications easier for parents and children alike, with positive effects for child well-being, including health (Asis, 2006; Nobles, 2011; Wen & Lin, 2012). It may be that phone and online interactions with children increase the chances that divorced parents stay engaged with specific aspects of children’s lives, including decisions concerning children’s health. Indeed, one study notes that children’s health is one of the most frequent subjects of conversation when Moldovan migrant parents, either married or divorced, communicate with family members back home (UNICEF, 2008). In this sense, the involvement patterns of divorced migrant parents may not be far from the involvement patterns of migrant parents who are in a stable marital relationship. More attention should be given in future research to specifics of parental involvement in child rearing when migration is marked by marital tensions.

The second contribution of this study is towards the analysis of different transnational forms of living in relation to children’s health. We found that regardless of the transnational setting, the overall relationship between migration and children’s health is positive or neutral. This finding suggests that more often than not, the benefits of migration overshadow the potential costs of separation. Such benefits are reflected in children’s health because this outcome is closely linked to household’s resources, as described by research in other contexts (Amuedo-Dorantes & Pozo, 2011; Donato & Duncan, 2011; Gerber & Torosyan, 2013; Stillman & McKenzie, 2012). However, depending on the specifics of transnational care, there are nuances on how migration relates to children’s health. In this study, children in returned migrant households, compared to children in non-migrant households, fared better or in no differing way in both countries. Return migration is beneficial for children because it encourages the transfer of norms and brings in additional human capital to the household (Lindstrom & Muñoz-Franco, 2006), especially when the returnee is the child’s primary caregiver (Vanore, 2015). Within the context of transnational migration, we also questioned whether the care arrangement linked to maternal and/or paternal absence is associated with decreased health for children. Other studies found more negative outcomes for children when mothers migrate (Cortés, 2007; Jordan & Graham, 2012; Parreñas, 2005; Schmalzbauer, 2004). Our findings run counter to these studies and show that maternal migration and the absence of both parents, when parents are in a stable marital relationship, do not result in poorer assessments of children’s health. It is important to note, however, that we specifically looked at who the caregiver of the child is when mothers and/or fathers migrate – something only a handful of studies have done before (e.g. Cebotari, Mazzucato, & Appiah, 2017; Mazzucao & Cebotari, 2017; Vanore, 2015). When fathers migrate and children are cared for by mothers, positive assessments of children’s health are found in Moldova and Georgia. When both parents migrate, data show that grandparents almost always assume the role of children's primary caregivers. Maternal and grandparental care was previously observed to play a key role in the coping process of Moldovan and Georgian children in that it was more likely to result in adequate nutritional intakes and proper access to healthcare services for children (Badurashvili & Nadareishvili, 2012; Robila, 2012; Vanore, 2015). Overall, our findings contradict the current narratives in Moldova and Georgia that characterize children of migrants as abandoned and instead emphasize ways in which household members stay engaged, as caregivers, with children back home. Policy initiatives that cater to children of migrants would do well to inform families on the advantages of care by a close family member and provide state subsidies for caregivers who stay in the country of origin.

This study also examines children’s reported health in relation to the duration of migration – a dimension that has proven important in transnational family research in other contexts (Donato & Duncan, 2011; Jordan & Graham, 2012; Vanore, 2015). Findings show that, over time, children in migrant households may develop resilience and adapt to changes in household configurations involving longer periods of migration. A long period of absence may indicate a stable labor residency abroad, better pay and stronger channels for remittances. These characteristics have been long praised by scholars for their potential to positively influence the development of children (Abrego, 2009; Amuedo-Dorantes & Pozo, 2011; Donato & Duncan, 2011; Gerber & Torosyan, 2013). Drawing on these studies, we also questioned whether the availability of remittances could be indeed a factor that positively associates with children’s health. Our findings show no differing health for children in migrant households compared to children in non-migrant households, when households receive remittances or not. This finding indicates that economic benefits from migration do not impact on the dimension of health measured here, and therefore provide no support to the claim that material gains from migration may be an influential factor in relation to children’s health in Moldova and Georgia. One plausible explanation for the absence of a remitting effect on reported health hinges on the social protection systems in the two countries. Specifically, both Moldova and Georgia provide free basic healthcare services to children, irrespective of the location of household members or the socio-economic status (Rukhadze, 2013; UNICEF, 2008). This largely reduces the need to pay for basic healthcare for children. Our data (not shown) support this assumption and reveal that only 1.3 and 1.4% of Georgian and Moldovan migrant households, respectively, had to use remittances to cover medical costs for children. Free access to vaccinations, doctors and basic medical facilities may level-up the general health of children in the country, rendering the effects of remittances insignificant. Another explanation relates to the remitting behavior of migrants with regard to health. Our data (not shown) indicate that 10.5 and 9.4% of Moldovan and Georgian migrant households, respectively, received medical provisions in cash or in-kind from migrants abroad. This evidence may suggest a contextualized remitting behavior, where households request and receive from migrants abroad medical provisions and additional cash when there is a medical emergency in the family. Because the analysis is based on cross-sectional data, we are unable to account for the time-varying component of this assumption. Future research involving panel data may shed light on the extent to which health outcomes benefit from migrants’ remitting behavior. Because our results may reflect the access to healthcare or a specific remitting behavior, or both, we caution readers to keep this in mind when assessing the results.

A third contribution of this study is to the transnational family literature in that we employ a gender perspective when measuring the health of children in transnational care. We find, similar to studies in other contexts (Cebotari, Mazzucato, & Appiah, 2017; Cortés, 2007; Gao et al., 2010; Antman, 2011), that Moldovan and Georgian girls in transnational households are less likely to have better reported health. The effects of various transnational characteristics and the size of the net relationships balance out in the two contexts, although the gender differentials seem to be slightly more evident in Georgia than in Moldova. Specifically, Moldovan girls in migrant households and girls with migrant fathers and cared for by mothers were more likely to have poorer reported health. In comparison, Georgian girls in migrant households, girls who were cared for by mothers when fathers were abroad, girls with household members remaining abroad for longer periods, and girls living in migrant households who received remittances were more likely to be assessed with poorer health. These findings merit special attention. Previous research has documented that both Moldova and Georgia are riddled by traditional gender norms that favor males (Badurashvili & Nadareishvili, 2012; UNICEF, 2008; Vanore, 2015). When family members migrate, households generally redistribute remaining work among those who stay behind. One study observed that Moldovan girls performed more physical chores at home than boys and also performed more chores when the family’s economic situation was improved by remittances (Cortés, 2007). Another study noted that Moldovan girls were “highly affected” by the extra physical work at home, compared with boys, who were only “moderately affected” by chores when family members migrate (UNICEF, 2008). In Georgia, Tchaidze and Torosyan (2010) revealed that females in migrant households were more likely than females in non-migrant households to assume traditionally male tasks which they may not have skills or the physical strength to perform properly. In addition, Georgian girls were found to be vulnerable to verbal abuse from caregivers, which subsequently affected their psychological health (Vanore, 2015). This evidence suggests that the relation between gender, migration and children’s health is not simple. Additional research is warranted to examine the particularities of gender with regard to health in these two contexts.

A final contribution of this study is towards the inclusion of two Eastern European countries to a body of literature dominated by research in Latin America and Asia. Overall, we find that children in transnational households are doing better or in a no differing way than children in non-migrant households. The consistency of results in both Moldova and Georgia suggest a wider regional pattern. What is especially noteworthy is that while we find advantages for children of migrants in Moldova and Georgia, there is also evidence of a more universal benefit from migration for children in a wider global context. Studies in Latin America (Amuedo-Dorantes & Pozo, 2011; Donato & Duncan, 2011; Frank, 2005; Lindstrom & Muñoz-Franco, 2006), Southeast Asia (Asis, 2006; Jordan & Graham, 2012) and Sub-Saharan Africa (Carling & Tønnessen, 2013; Cebotari, Mazzucato, & Appiah, 2017; Yabiku et al., 2012) found contextually-based evidence that children in transnational care are not necessarily disadvantaged in health outcomes when family members migrate. Yet, under certain circumstances, as this study shows, girls in transnational care may have poorer health compared to boys. This evidence is indicative of the hardship and loss that some children may experience when family members migrate (Dreby, 2010; Parreñas, 2005; Schmalzbauer, 2004; Wen & Lin, 2012), although only a handful of studies were able to distinguish the results by gender (Antman, 2011; Cortés, 2007; Gao et al., 2010). To the extent this study is suggestive of a differential health effect of migration by gender, more cross-regional evidence from matching data sources is needed.

This study is not without limitations. One limitation concerns the cross-sectional nature of our data. Because data capture snapshot reports, the results imply correlations and not causation, and we caution readers to consider this difference when assessing the findings. Another limitation relates to the use of caregiver-reported subjective health. Previous studies found that caregivers sometimes under-report children’s outcomes (Cebotari, Siegel, & Mazzucato, 2016; Jordan & Graham, 2012). Future research is advised to include two comparable sets of questions to be answered by both caregivers and children to increase the reliability of assessments. A final limitation concerns the complexity of factors when analyzing comparative data. Despite employing a number of different transnational characteristics, we could not identify all factors that describe the life of children in transnational care in the two countries. It was thus not possible to conduct analysis on specifics of migration such as multiple migration spans, the frequency of contact, the country of destination, children’s ages at separation, the type of employment, or the residency status abroad. Future analyses must detail these factors in relation to children’s health.

Despite these limitations, this study is a rare opportunity to consider how children in origin countries experience transnational migration with population-representative evidence. Overall, the findings suggest that children’s health is more sensitive to proximate characteristics such as gender than it is, perhaps, to migration. Future research based on different transnational factors and employing longitudinal data in different contexts will be helpful to further test this line of inquiry.
